# Antimicrobial Nanomaterials in the Food Industry: Applications in Meat Packaging

**DOI:** 10.3390/ma19061160

**Published:** 2026-03-16

**Authors:** Catalina-Elena Constantin, Alina Maria Holban, Florin Iordache, Carmen Curutiu

**Affiliations:** 1 Department of Microbiology and Immunology, Faculty of Biology, University of Bucharest, 030018 Bucharest, Romania; catalina-elena.butaru@s.unibuc.ro (C.-E.C.); carmen.curutiu@bio.unibuc.ro (C.C.); 2 Microbiology Laboratory, TÜV Austria Romania, Calea Plevnei nr.139 B, Corp A, Sector 6, 060011 Bucharest, Romania; 3 Research Institute of the University of Bucharest, University of Bucharest, 030018 Bucharest, Romania; 4 Faculty of Veterinary Medicine of Bucharest, University of Agronomic Sciences and Veterinary Medicine of Bucharest, 105 Splaiul Independentei, 050097 Bucharest, Romania; floriniordache84@yahoo.com

**Keywords:** antimicrobial nanomaterials, meat packaging, active packaging, food safety, zinc oxide nanoparticles, migration, green synthesis

## Abstract

A thorough understanding of the microbial ecology of meat products, dominated by critical pathogens such as *Salmonella* spp., *Campylobacter jejuni*, *Escherichia coli*, and *Listeria monocytogenes*, and marked by risks of resistant biofilm formation and vulnerabilities specific to informal commercial sectors, underscores the need to transition from conventional inert barriers to active nanostructured packaging systems. This review critically analyses the current state of antimicrobial nanomaterials, dissecting their molecular mechanisms of action and dynamic interactions designed to preserve sensory and nutritional food quality. Beyond technical effectiveness, the paper highlights the inherent tension between technological innovation and toxicological uncertainties, addressing major challenges related to migration kinetics in complex lipid matrices and the uneven global regulatory landscape. Main limitations of frequently investigated materials, along with regulatory discrepancies among international authorities and safety variables, are discussed to contextualise the current barriers to industrial implementation. We conclude that although nanotechnology represents a transformative force for extending shelf life, safety validation through rigorous assessment of migration remains imperative to harmonise scientific progress with public health protection. This integrative perspective highlights the imperative of calibrating nanostructural architecture to the bioactive profile, providing strategic design directions essential for the sustainable translation of experimental innovation to industrial scale.

## 1. Introduction

In a global context marked by population growth and increasing pressure on resources, the food industry faces the major challenge of ensuring the safety and quality of perishable products. Safety and the sustainability of supply chains are fundamental pillars of contemporary society in a context where food waste and foodborne diseases continue to generate immense economic and social costs. Meat and meat products are ideal nutrient media for microbial proliferation, characterised by high water activity (aw), neutral pH and an abundance of oxidisable proteins and lipids. These biochemical characteristics make meat products extremely susceptible to rapid oxidative degradation, colonisation by pathogenic microorganisms and spoilage, which requires the development of barrier technologies superior to conventional methods [1].

Conventional preservation and packaging methods, although useful, have reached their limits in meeting modern demands for fresh, minimally processed and safe food [1]. In this landscape, nanotechnology has emerged as a transformative force, offering innovative solutions through the development of active, intelligent packaging that dynamically interacts with products and their environments. Traditionally, packaging systems have functioned primarily as passive, inert barriers, isolating the product from the external environment to prevent secondary contamination and dehydration. Nevertheless, a critical examination of recent comprehensive reviews [2,3], underscores the inability of these classic packaging materials to respond dynamically to changes in the package’s internal environment or to actively intervene in biological degradation processes. In this context, nanotechnology has emerged as a transformative frontier, facilitating a paradigm shift from passive packaging to active, intelligent systems.

The concept of active packaging goes beyond the traditional function of an inert barrier, involving deliberate interaction between the packaging, the product and the environment to extend shelf life and maintain sensory and nutritional quality. As elucidated in recent authoritative studies focused on shelf-life extension mechanisms [1], the operational capability of these systems can act either by absorbing unwanted substances (oxygen, moisture, and ethylene) or by the controlled release of antimicrobial and antioxidant agents into the headspace or directly onto the food surface. Conversely, the domain of innovative packaging, as extensively differentiated in foundational literature [4] regarding functional materials, emphasises a critical distinction between active packaging, which releases or absorbs substances to extend shelf life, and innovative packaging, which monitors and communicates the product’s condition (e.g., time temperature indicators or freshness sensors).

Food nanotechnology involves manipulating matter at the atomic and molecular scales to create materials with distinct physicochemical properties. It is postulated within the field of nanobiotechnology [5] that this approach possesses the transformative potential to revolutionise the industrial landscape by significantly augmenting gas and moisture barrier properties, enhancing mechanical structural integrity, and introducing intrinsic antimicrobial functionalities. From the use of nanoclays to reinforce polymers to the integration of metal nanoparticles (silver, zinc, and titanium) to combat pathogens, the range of applications is vast.

Nanomaterials, defined as materials with dimensions in the 1–100 nm range, have an exceptional surface-to-volume ratio, giving them chemical and biological reactivity far superior to that of their macroscopic equivalents. Recent analytical frameworks regarding nanomaterial integration suggest [6] that integrating nanoparticles into packaging polymers enables a radical modification of mechanical (tensile strength and flexibility), thermal, and gas barrier properties. Furthermore, these materials can confer novel bioactive functionalities, such as intrinsic antimicrobial activity, ethylene absorption capacity, and UV radiation blocking, which are essential for preventing lipid oxidation and maintaining organoleptic quality.

**Figure 1:**
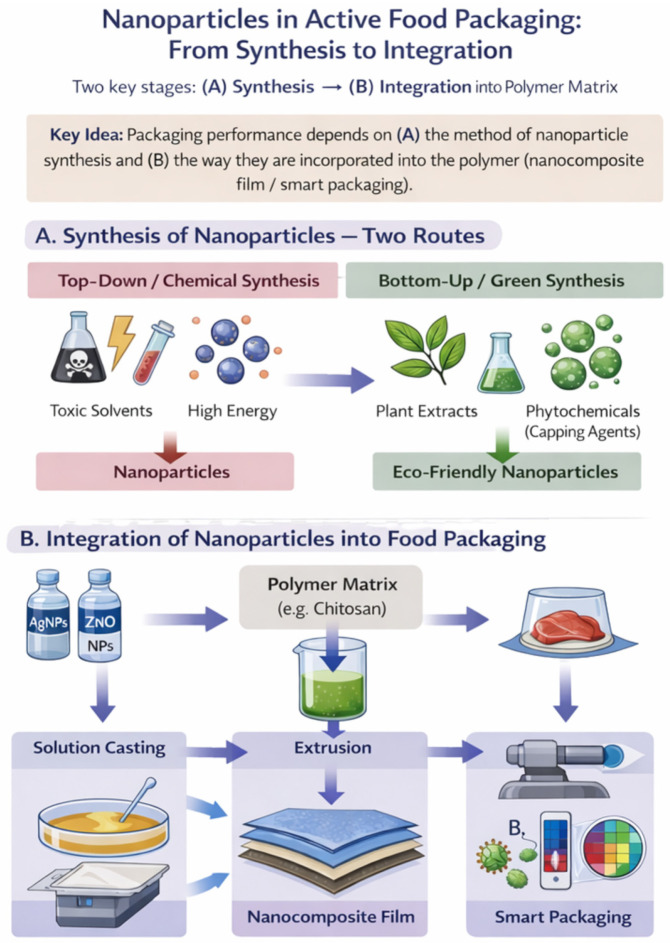
Conceptual workflow illustrating the deployment of nanoparticles in active food packaging systems, from nanomaterial production to functional incorporation within polymeric matrices. (**A**) Two principal synthesis pathways are depicted: conventional top-down/chemical routes, which may entail elevated energy demand and the use of hazardous solvents or reagents, and bottom-up “green” synthesis, in which plant extracts supply reducing and capping constituents that govern nucleation, growth kinetics, and colloidal stability, yielding nanoparticles with a comparatively improved environmental profile. (**B**) Nanoparticle integration (e.g., AgNPs and ZnO NPs) into a polymer matrix (e.g., chitosan) is schematised via representative processing strategies—solution casting and extrusion—through which nanofiller dispersion and interfacial interactions are tuned, thereby determining the resulting material architecture and performance. The downstream outputs include nanocomposite films for active packaging (enhanced barrier functionality and antimicrobial/antioxidant activity mediated by material-dependent contact effects and/or controlled release) and smart packaging formats, wherein nanomaterials serve as sensing or indicating elements (e.g., colourimetric or electrochemical responses) to support in situ monitoring of product quality throughout refrigerated storage and distribution (created in BioRender.com, accessed on 5 February 2026).

Among the nanomaterials investigated, silver nanoparticles (AgNPs) are considered the “gold standard” in current research due to their remarkable thermal stability and broad-spectrum biocidal activity. Current comprehensive reviews on silver nanoparticles [7] posit that these agents exert their effects through a multifaceted mode of action, which includes the generation of reactive oxygen species (ROS) and interference with bacterial DNA replication, thereby proving effective even against multidrug-resistant strains. However, their use raises serious issues of toxicity and ecological impact. This duality has stimulated research into green synthesis methods, which use plant or microbial extracts as reducing and stabilising agents, offering a biocompatible alternative to conventional chemical synthesis. However, the transition to these advanced technologies requires a thorough understanding of the interactions between nanomaterials and the food matrix. It is strongly argued in recent advancements in sustainable nanotechnology [8] that the deployment of biodegradable biopolymers, such as polylactic acid (PLA) or chitosan, reinforced with nanostructures, represents the prospective trajectory for mitigating the environmental impact of conventional plastics. Biopolymers often have limitations in terms of mechanical and barrier properties. Recent investigations into bionanocomposites [9,10] contend that reinforcing these matrices with nanofillers (metal nanoparticles and nanoclays) is the optimal approach to creating nanocomposites with superior performance. Furthermore, the synthesis of these nanomaterials has evolved towards “green chemistry” methods, using plant extracts, fungi (mycofabrication) or bacteria to reduce toxicity and environmental impact (Figure 1).

In this advanced materials landscape, zinc oxide nanoparticles (ZnO NPs) have emerged as a pivotal element in active packaging design, distinguished by their intrinsic multifunctionality that combines potent antimicrobial efficacy with exceptional UV-barrier properties [11,12]. Consequently, these nanostructures have become the subject of intense investigation for meat preservation strategies, offering a biocompatible mechanism to arrest microbial proliferation while preserving the physicochemical integrity of lipid-rich matrices [13,14].

This paper aims to critically analyse the current state of antimicrobial nanomaterials in the meat industry, highlighting limitations and challenges of most frequently investigated materials, offering a perspective on their impact on further research. Here, we also dissect their molecular mechanisms of action, summarise recent applications on various meat matrices and discuss significant regulatory challenges, in particular the phenomenon of migration and legislative discrepancies between international authorities.

## 2. Microbial Contamination of Meat Products: Targets and Detection Strategies

Understanding the microbial ecology of meat products is pivotal for the rational design of effective packaging systems. Meat constitutes a highly permissive substrate for microbial colonisation owing to its nutrient density and elevated water activity, and it may be contaminated at multiple nodes along the value chain, including slaughter, processing, and distribution. Consequently, microbiological control necessitates integrated interventions that extend beyond refrigeration alone. Among the principal hazards, *Salmonella* spp., *Campylobacter jejuni*, *Escherichia coli*, and *Listeria monocytogenes* continue to represent major public-health concerns. Global surveillance and epidemiological datasets [15,16] consistently implicate poultry meat as a significant vehicle for campylobacteriosis and salmonellosis transmission worldwide.

In low- and middle-income settings, the epidemiology of meatborne contamination is further conditioned by the pronounced structural heterogeneity between formal (regulated, inspected slaughter, and distribution systems) and informal (backyard slaughtering and street-vended meat) sectors. Comparative evaluations of meat safety governance and practice [17] indicate that informal supply chains are frequently characterised by absent or inconsistent veterinary and sanitary oversight, limited cold chain capacity, and suboptimal hygienic practices, including slaughter on contaminated ground surfaces and restricted access to potable water. These constraints collectively amplify opportunities for microbial proliferation and cross-contamination, resulting in carcasses with elevated bacterial loads and recurrent detection of enteric pathogens such as *E. coli* and *Salmonella*, particularly under conditions of prolonged ambient temperature exposure and inadequate handling controls. Conversely, although the formal sector operates within regulatory frameworks, it remains vulnerable to contamination at critical processing points and to cold chain disruptions, as documented in studies examining bioaerosol dynamics and carcass contamination patterns [17]. Importantly, the associated risk is not confined to episodic failures in process hygiene: these organisms can persist within processing environments through the establishment of resilient biofilms on equipment and contact surfaces, thereby diminishing the effectiveness of conventional disinfection regimens and sustaining contamination pressure over time.

From a translational and implementation perspective, the adoption of advanced antimicrobial nanocomposite packaging within informal meat supply chains must be evaluated in relation to economic feasibility, manufacturing scalability, and infrastructural limitations. Although nanocomposite-based packaging materials exhibit enhanced barrier performance and antimicrobial activity, their large-scale implementation often requires sophisticated production technologies, regulatory approval pathways, and controlled processing environments, which may limit rapid deployment in decentralised or resource-constrained markets [18,19,20]. Multilayer nanocomposite films often require co-extrusion or solvent casting technologies that are rarely available in decentralised meat markets. In contrast, bio-based edible coatings formulated from natural polymers such as chitosan, alginate, starch, or protein matrices constitute a more feasible intervention for informal sectors, as these materials can be sourced from renewable feedstocks, applied using simple techniques (e.g., dipping or spraying), and integrated into existing meat handling practices without substantial capital investment [21]. Moreover, such edible coatings can act as carriers of natural antimicrobial agents, including essential oils and plant-derived compounds, thereby contributing to microbial inhibition and shelf-life extension while maintaining environmental sustainability [21,22]. Accordingly, a tiered implementation strategy may be envisaged, whereby technologically sophisticated nanocomposite packaging is primarily deployed in formal industrial processing systems, whereas cost-effective edible or biodegradable coatings provide a realistic and scalable approach for mitigating microbial contamination in informal meat markets. This differentiated deployment framework aligns advanced packaging innovation with socio-economic feasibility and enhances the translational relevance of antimicrobial packaging solutions across heterogeneous meat supply chains [18,22].

### 2.1. Pathogen Spectrum and Spoilage Microbiota

Recent literature identifies fresh meat, especially poultry, as a primary vector for major foodborne pathogens [16]. In order to help limit their spread, nanotechnology specifically targets the following groups:

#### 2.1.1. *Campylobacter jejuni*

This microaerophilic pathogen is commonly associated with raw chicken meat and is a significant cause of human gastroenteritis. National-scale surveillance conducted in Belgium [23] has demonstrated that the prevalence of *Campylobacter* in chicken meat preparations varies considerably between companies, highlighting the need for more robust food safety management systems (FSMS) capable of controlling raw materials with high microbial loads. Recent experimental studies [24] have demonstrated that active packaging systems functionalised with immobilised zinc oxide (ZnO) nanoparticles are highly effective. The mechanism involves inducing severe oxidative stress on the bacterial cell membrane, thereby significantly reducing the pathogen load in meat exudates without compromising sensory quality.

#### 2.1.2. *Salmonella* spp.

Along with *Campylobacter*, *Salmonella* remains a critical target. Literature concerning biofilm mitigation strategies [25,26] highlights the necessity for nanocomposites capable of preventing *Salmonella* biofilm formation on meat surfaces. Biofilms confer resistance to disinfectants, but metal nanoparticles can penetrate the exopolysaccharide matrix, neutralising the protected bacteria.

#### 2.1.3. Spoilage Microbiota (Psychrotrophs)

Beyond pathogenic risks, economic degradation is caused by psychrotrophic bacteria (*Pseudomonas* spp. and *Brochothrix thermosphacta*), yeasts, and moulds. These microorganisms are responsible for organoleptic degradation via proteolysis and lipolysis, resulting in substantial economic losses [1,27]. It is emphasised in recent reviews on biodegradable polymers [28] that nanostructured packaging must offer broad-spectrum activity to suppress this competitive microbiota and extend commercial shelf life. Because psychrotrophic bacteria are major drivers of spoilage during chilled storage, nanomaterial-enabled packaging is also relevant for controlling spoilage microbiota under refrigeration conditions [29]. In particular, Ag- and ZnO-based nanostructures can exert antimicrobial effects at the food packaging interface by disrupting membrane integrity/permeability and promoting oxidative stress via reactive oxygen species (ROS), with additional contributions from released metal ions (Ag^+^ or Zn^2+^) that intensify cellular damage [30,31]. Moreover, active coatings incorporating ZnO nanoparticles have been reported to inhibit psychrotrophic bacterial growth during refrigerated storage in packaged foods, supporting their applicability beyond classical pathogen control [32].

### 2.2. Advanced Detection Methods: From Classical Culture to Nanosensors

**Figure 2:**
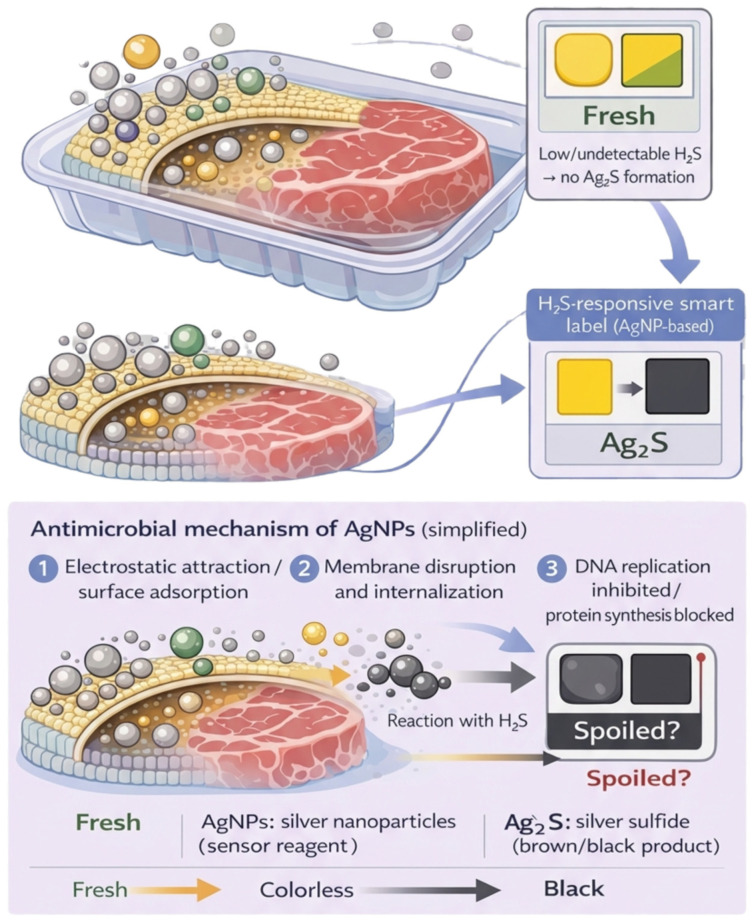
Architecture of intelligent packaging systems for meat: freshness monitoring and antimicrobial action. The smart label is an H_2_S-responsive indicator in which silver nanoparticles (AgNPs) are immobilised within a polymeric/porous matrix. For fresh meat, spoilage-related volatile sulphur compounds are low or undetectable, and the label remains yellow (no conversion of AgNPs). As microbial spoilage advances, bacteria produce hydrogen sulphide (H_2_S) and related sulphur volatiles; H_2_S reacts with AgNPs to form silver sulphide (Ag_2_S), a brown-to-black product, resulting in a visible colour change that indicates potential spoilage. The bottom scheme summarises the colour transition (yellow → colourless/dimmed → black) associated with the AgNPs → Ag_2_S conversion. The central numbered pathway depicts a simplified antimicrobial action of AgNPs: (1) electrostatic attraction and adsorption onto the microbial cell surface, (2) membrane disruption and internalisation, and (3) inhibition of DNA replication and blockage of protein synthesis (created in BioRender.com, accessed on 5 February 2026).

In the modern paradigm, packaging must not only protect but also communicate. The importance of real-time monitoring of microbial load for safety validation is strongly advocated in studies evaluating food safety management systems [33]. Contamination monitoring has evolved from conventional microbiological methods, which are laborious and time-consuming (24–72 h), to rapid nanotechnology-based solutions. Nanotechnology allows colourimetric sensors to be integrated directly into the packaging structure (Figure 2). Developments in nanometric sensors capable of detecting specific spoilage metabolites (biogenic amines and volatile sulphur compounds) or even the presence of bacterial DNA have been extensively described [4].

For example, gold (AuNPs) and silver (AgNPs) nanoparticles functionalised with antibodies or aptamers can be used for rapid colourimetric detection of *Salmonella* or *E. coli*. Recent advances [8] describe the use of 60 nm silica (SiO_2_) nanoparticles doped with fluorescent molecules for the rapid detection of *E. coli* O157:H7, offering much higher sensitivity than classical methods. Similarly, [15] potential applications of nanosensors for real-time detection of contaminants in slaughterhouse cooling water, allowing automatic adjustment of biocide doses, have been discussed. Sensors based on silver nanoparticles (AgNPs) that visually detect volatile spoilage compounds are also described in current literature [7]. For example, in a specific study [34], utilising hydrogel matrices, a Gellan gum-based hydrogel incorporated with AgNPs was used to monitor chicken and fish meat. In the presence of hydrogen sulphide (H_2_S)—a metabolite produced by proteolytic spoilage bacteria—the silver nanoparticles undergo a chemical reaction, transforming into silver sulphide, which causes a colour change from yellow to colourless or brown. This colour transition provides the consumer with a visual, non-invasive and irreversible indication of freshness (Table 1), directly correlated with the increase in the total viable bacteria count (TVC).

## 3. Food Packaging Regulations: Safety and Migration

The commercial implementation of nanomaterials in food packaging is a double-edged sword: technological innovation clashes with toxicological uncertainties, creating a complex, uneven global legislative landscape.

### 3.1. Relevance of Regulations for Meat Products

When assessing the safety of nanostructured packaging intended for meat, regulatory compliance hinges on whether migration is demonstrated to remain acceptable under credible worst-case conditions relevant to the product category. Systematic reviews of recent literature highlight that migration kinetics in nano-enabled systems are strongly context-dependent; therefore, compliance-orientated testing must be parameterised using variables that reflect realistic (and, where appropriate, conservative) use scenarios [14,36]. For meat products, the compliance-relevant variables can be grouped into three domains: the physicochemical nature of the food matrix; the time temperature profile over storage and distribution; and multilayer architectures, including the effectiveness of any functional barrier separating active layers from the food contact surface [7,14].

#### 3.1.1. Nature of the Food (Fatty Matrix)

Fat-rich meat matrices do not act merely as passive recipients of migrating species but can actively modulate the mass transfer behaviour of packaging constituents through multiple physicochemical mechanisms. While diffusion remains the dominant transport process governing the migration of low-molecular-weight additives and released metal ions, lipid-rich environments may additionally induce partial swelling and plasticisation of polymeric matrices, thereby increasing chain mobility and facilitating the release of embedded nano-enabled agents. Such interactions have been described for common food contact polymers, where the sorption of lipophilic compounds into amorphous regions of the polymer can increase free volume and enhance molecular diffusion rates during prolonged contact with fatty simulants or real food matrices [37,38].

Experimental investigations have demonstrated that fatty simulants (e.g., ethanol-based or oil-rich media) can significantly increase apparent diffusion coefficients compared to aqueous systems, reflecting combined effects of higher solubility of migrants and partial relaxation of the polymer network [37]. This behaviour suggests that migration in fatty foods is not governed solely by classical Fickian diffusion but may involve coupled sorption–diffusion mechanisms in which lipid uptake modifies the intrinsic transport properties of the packaging material [38,39]. In this context, diffusion coefficients of typical organic migrants in polyolefin matrices have been reported to increase by up to one order of magnitude in fatty environments relative to water-based media, highlighting the role of polymer–lipid interactions in accelerating mass transfer [37]. Recent experimental studies have reported diffusion coefficients of organic additives in polymer–food simulant systems in the range of ~10^−15^ to 10^−12^ m^2^/s, with higher values observed in ethanol-based or lipid-like simulants due to polymer swelling and enhanced partitioning effects [40].

For nano-enabled antimicrobial systems, these effects may be further amplified by interfacial interactions between lipids, proteins, and the polymer matrix, which can destabilise nanoparticle–polymer interactions and promote the release of ionic species rather than intact particles. Studies addressing nanoparticle behaviour in food contact plastics indicate that lipid-rich environments can enhance mobility through localised matrix plasticisation and microstructural rearrangements, although the magnitude of this effect strongly depends on nanoparticle immobilisation, polymer crystallinity, and storage temperature [39,41]. Consequently, migration in fatty meat products should be interpreted as a combined mechanism involving diffusion-controlled transport, lipid-induced polymer swelling, and partition-driven release at the packaging–food interface.

These mechanistic considerations support the use of fatty food simulants (e.g., 50% ethanol or simulant D2) in migration testing protocols, as recommended in regulatory guidelines, since such simulants better reproduce the sorption capacity and plasticising effects exerted by real meat matrices on polymeric packaging systems [37,38].

#### 3.1.2. Storage Conditions

Meat products frequently undergo prolonged refrigeration or freezing. Temperature fluctuations and freeze–thaw cycles may induce polymer chain relaxation and microstructural changes that alter porosity and diffusion pathways, thereby affecting the effective diffusion rate of nanoparticles and/or their transformation products [1,6].

#### 3.1.3. Multilayer Systems

Meat is frequently packaged in multilayer structures designed to achieve high gas-barrier performance and mechanical stability, enabling the protection of perishable matrices against oxygen, moisture, and microbial contamination. In such systems, regulatory frameworks require a rigorous evaluation of the so-called functional barrier, defined as the layer that prevents the migration of active or non-authorised substances from inner layers into the food contact surface beyond acceptable limits [7]. This requirement is explicitly established within European legislation governing plastic materials and articles intended to come into contact with food, which mandates that both overall and specific migration of components be assessed under realistic conditions of use to ensure consumer safety [42].

The effectiveness of multilayer functional barriers is strongly influenced by the physicochemical properties of the polymeric constituents, including polymer composition, crystallinity, layer thickness, and interfacial adhesion between adjacent layers. These parameters collectively modulate diffusion pathways and determine the extent to which nano-enabled antimicrobial agents or their transformation products may be released from internal layers into complex food matrices such as fresh meat, characterised by high lipid and protein contents. From a mass transfer perspective, multilayer architectures introduce additional resistance to diffusion; however, imperfect dispersion, polymer relaxation phenomena, and temperature-dependent changes in free volume may still facilitate limited migration under prolonged storage or thermal fluctuations [43].

Consequently, matrix-related factors (e.g., fat content and protein interactions), storage conditions (temperature and time), and structural characteristics of the packaging system (layer arrangement and barrier efficiency) must be considered jointly when predicting the migration behaviour and exposure potential of nanomaterials used in antimicrobial meat packaging applications. These interdependent variables underscore the need for a comprehensive evaluation of multilayer nanocomposite systems within established regulatory risk-assessment frameworks for nano-enabled food contact materials [42,43].

#### 3.1.4. Regulatory Risk-Assessment Frameworks (EFSA and FDA Perspectives)

Within this broader context, the safety evaluation of antimicrobial nanomaterials intended for meat packaging must be interpreted in light of internationally recognised regulatory risk-assessment frameworks, particularly those developed by the European Food Safety Authority (EFSA) and the U.S. Food and Drug Administration (FDA) [44,45].

From a regulatory science perspective, these official guidance documents emphasise that nano-enabled food contact materials cannot be assessed solely on the basis of the toxicological profile of their bulk chemical equivalents, as nanoscale properties may significantly modify reactivity, dissolution behaviour, and biological interactions following ingestion or migration into food matrices [44,45]. Consequently, comprehensive physicochemical characterisation is required, including particle size distribution, surface chemistry, agglomeration state, and solubility under realistic conditions of use.

The EFSA framework explicitly highlights the need for migration testing under realistic and worst-case scenarios, particularly for lipid-rich foods such as meat products, where protein–lipid interactions and storage conditions may influence nanoparticle partitioning and release kinetics at the packaging–food interface. In this regard, EFSA adopts a precautionary, case-by-case evaluation approach, requiring separate authorisation for substances engineered at the nanoscale even when their conventional forms are already approved [44].

In contrast, FDA guidance adopts a substance-orientated yet exposure-driven assessment philosophy, focusing on intended use, estimated dietary exposure, and potential physicochemical transformations of nanomaterials during processing and storage. This framework recognises that nano-enabled food contact substances may undergo structural and chemical changes that alter migration behaviour and systemic exposure, thereby necessitating a comprehensive safety evaluation integrating material characterisation, release potential, and toxicological endpoints [45].

The relevance of these regulatory principles is further supported by primary experimental studies conducted directly on meat matrices. For example, antimicrobial gelatin films incorporated with silver nanoparticles have been shown to significantly extend the shelf life of refrigerated fish fillets while maintaining migration levels within established safety thresholds, highlighting the importance of nanoparticle immobilisation within the polymer network [46]. Similarly, immobilised ZnO nanoparticle active packaging systems have been reported to effectively reduce *Campylobacter jejuni* contamination in raw chicken meat without compromising sensory quality, confirming that nanoparticle dispersion and polymer matrix interactions are critical determinants of both antimicrobial efficacy and regulatory safety [24].

Collectively, integrating authoritative regulatory guidance documents with representative primary experimental investigations provides a scientifically robust framework for assessing nano-enabled antimicrobial packaging in meat systems. Such an approach reduces reliance on generalised conclusions derived exclusively from secondary review literature and ensures that technological claims are grounded in experimentally validated evidence aligned with internationally accepted food safety governance principles [44,45].

Within this regulatory landscape, differences between major authorities become particularly relevant, as EFSA’s precautionary, nano-specific authorisation model contrasts with the more substance-based and exposure-driven evaluation philosophy adopted by the FDA. While these frameworks share common scientific objectives related to migration assessment and consumer safety, their regulatory implementation and authorisation philosophies differ substantially, as discussed in the following section [44,45].

### 3.2. Migration Phenomenon and Toxicological Risk

The main barrier to widespread adoption is migration, the transfer of nanoparticles and/or their transformation products from the polymer matrix into the meat product. Therefore, migration (e.g., 50% ethanol or vegetable oil—simulant D2) is perferred as aqueous simulants may underestimate the migration rate of active compounds [14,47]. Moreover, it is noted that the acidity of meat products (e.g., marinades) can facilitate the dissolution and release of metal ions from metal nanoparticles (e.g., Ag and ZnO) [48]. Controlled release systems (CRPs) must be designed so that the release kinetics of the antimicrobial agent are synchronised with the growth rate of the target microorganisms (lag phase), as discussed in specialised literature [47]. Too rapid release can lead to depletion of the agent before the end of its shelf life, while too slow release can allow initial bacterial proliferation. Attention is drawn [6] to the “large surface area” paradox: the huge surface-to-volume ratio that gives nanoparticles their antimicrobial effectiveness simultaneously increases their biological reactivity towards human cells. Once ingested through contaminated food, nanoparticles can cross biological barriers (intestinal and blood-brain) and accumulate in vital organs (liver and kidneys), inducing oxidative stress, cytotoxicity and potential genotoxicity. Studies cited in toxicological assessments [12,49] highlight the absolute necessity of assessing the potential for long-term bioaccumulation.

Importantly, migration-related safety is not the only barrier to widespread adoption. Practical implementation is also constrained by economic, environmental, and societal factors. From a commercialisation standpoint, reviews of active and nano-enabled packaging repeatedly note that scaling from laboratory formulations to manufacturable materials can involve high raw-material costs (particularly for certain nanomaterials), additional process controls, and increased validation and regulatory testing burdens, all of which can raise unit costs and slow technology transfer [50,51]. Environmental considerations introduce further constraints: life-cycle-based analyses of nanomaterials and packaging systems indicate that production routes, energy intensity, and end-of-life scenarios can dominate environmental hotspots, and they highlight the need to evaluate whether shelf-life gains outweigh added impacts under realistic waste and disposal assumptions [52,53]. Finally, consumer acceptance remains decisive for market uptake: empirical studies show that willingness to buy nano-enabled applications is strongly shaped by perceived benefits, perceived risks, and trust, and that acceptance can vary substantially depending on whether nanotechnology is used in packaging rather than directly in food [54].

### 3.3. Legislative Divergences: FDA vs. EFSA

There is an apparent dichotomy between regulatory approaches in the United States and the European Union, described in sources as a “regulatory dilemma”. Significant differences have been highlighted in comparative regulatory reviews [1,48].

- FDA (United States): The Food and Drug Administration takes a more pragmatic stance, focusing on the evaluation of the base chemical. Thus, materials such as zinc oxide (ZnO) and certain silver salts are recognised as GRAS (Generally Recognised As Safe) and have received approvals for use in contact with food, being considered biocompatible within certain concentration limits [3]. This encourages rapid innovation but raises questions about the long-term effects of exposure to nanoforms.
- EFSA (European Union): The European authority maintains a strict precautionary stance. Nanomaterials require a “case-by-case” risk assessment. According to legislative analyses of market requirements [55] and recent comprehensive reviews [7], EU legislation (Regulation (EU) No 10/2011) [42] requires substances in “nano” form to be explicitly authorised, even if their macroscopic form is already approved. EFSA requires detailed physicochemical characterisations and imposes extremely low Specific Migration Limits (SMLs) (e.g., 0.05 mg/kg for silver in specific contexts). The necessity for advanced analytical methods (such as ICP-MS coupled with fractionation) to detect and characterise nanoparticles that could migrate into meat products is highlighted, ensuring compliance with these strict limits [10,56]. A concise comparison of specific migration limits and authorisation approaches for Ag and ZnO in the EU and USA is provided in Table 2.

This rigour requires standardised safety validation protocols, which slows down the market introduction of innovations but guarantees a higher level of consumer protection.

## 4. Nanomaterials for Antimicrobial Packaging: Mechanisms, Innovations and Mirror Comparison

Nanomaterials are revolutionising antimicrobial packaging through mechanisms of action that target the fundamental microbial structures. They enable surface-mediated (“contact-active”) effects and/or controlled-release functionalities that are difficult to replicate with conventional additives. Their effectiveness derives from their small size (1–100 nm) and superior surface reactivity [60]. Nevertheless, translation from laboratory efficacy to industrial deployment is not determined by antimicrobial potency alone but by a mirror appraisal that weighs performance under realistic meat matrices against safety/regulatory burden, technoeconomic scalability, environmental footprint, and consumer acceptance.

The following tables summarise the literature data, organised by two key perspectives: the material used (Table 3) and the target microorganism (Table 4).

### 4.1. Metallic Nanoparticles

#### 4.1.1. Silver (AgNPs): Highest Antimicrobial Breadth, Highest Scrutiny

It is the dominant antimicrobial agent in research, recognised for its effectiveness against Gram-positive and Gram-negative bacteria. A broad spectrum of action that includes membrane adhesion, cell penetration, and the generation of reactive oxygen species (ROS) has been described [2,65]. The molecular mechanisms detailed in recent comprehensive reviews [7,66] include the following: (1) generation of reactive oxygen species (ROS) that induce lethal oxidative stress; (2) disruption of cell membrane integrity through pore formation; (3) release of Ag^+^ ions that penetrate the cytoplasm, bind to the thiol groups of respiratory enzymes, and interact with DNA, blocking replication. A crucial innovation is green synthesis, discussed in recent studies on nanocomposites [7,20], which uses plant extracts to reduce residual toxicity and increase biocompatibility. From a „mirror” perspective, their key advantage is robust antimicrobial activity that can be formulated into coatings or nanocomposites; the principal disadvantages are a comparatively high cost base (material price, dispersion control, and analytical validation) and a higher regulatory and toxicological scrutiny due to the need for nanospecific characterisation and exposure assessment in food contact uses [67]. Consequently, AgNP-based packaging is often best positioned for designs that minimise potential exposure (e.g., immobilised/contact-active architectures) while preserving antimicrobial performance.

#### 4.1.2. Zinc Oxide (ZnO)

It is valuable for meat products due to its multifunctionality. It has been demonstrated that ZnO NPs biosynthesised with Lactobacillus plantarum have an oval shape and an average size of 29.7 nm, being extremely effective against *Salmonella*, *E. coli* and *S. aureus* [13]. The primary mechanisms identified are ROS-mediated oxidative stress and leakage of cellular contents (proteins, sugars) resulting from membrane damage. Studies on essential oil-based systems highlight its ability to block UV radiation, preventing photochemical oxidation of lipids [11]. Antimicrobial, the photocatalytic mechanism by which ZnO generates hydroxyl radicals and superoxide anions under illumination, destroying the bacterial cell wall, has been elucidated [12]. The „mirror” limitation is that ZnO efficacy and Zn^2+^ release kinetics can be strongly matrix- and pH-dependent: proteins, salts, and lipids in real meat exudates may attenuate activity relative to in vitro tests, while acidic conditions may accelerate dissolution beyond controlled-release targets [68]. Thus, ZnO is frequently a more scalable and cost-favourable platform than AgNPs, but it requires matrix-aware optimisation (polymer selection, dispersion state, and release control) to ensure consistent performance [12].

#### 4.1.3. Other Materials (TiO_2_ and CuO)

Titanium dioxide and copper oxide are being explored for their photocatalytic and antifungal properties, being effective in illuminated environments [69,70].

### 4.2. Nanostructured Biopolymers: Functional Matrices

Biopolymers such as chitosan, pectin and pullulan serve as vehicles for nanoparticles, forming nanocomposites with improved properties. Sustainability is addressed by replacing synthetic plastic with functionalised biopolymers.

#### 4.2.1. Nanocellulose

Cellulose nanocrystals (CNCs) mechanically reinforce films and act as carriers for the controlled release of active agents (Ag and ZnO). Recent experimental validations concerning cellulosic reinforcements have demonstrated that these nanocomposites significantly augment the oxygen barrier integrity and thermal stability of the packaging matrix, thereby confirming their utility in advanced preservation systems [62,63]. In addition to cellulose nanocrystals (CNCs), other nanocellulose forms—particularly cellulose nanofibrils (CNFs) and bacterial cellulose (BC)—have also been explored for food contact applications. CNFs, owing to their high aspect ratio and fibrillar entanglement network, can provide strong reinforcement and can improve barrier performance through tortuous diffusion pathways; however, their pronounced hydrophilicity may increase moisture sensitivity unless combined with hydrophobic layers or crosslinking strategies. Bacterial cellulose, produced by microbial fermentation, offers a highly pure, highly crystalline nanofibrillar structure with excellent mechanical integrity and water-holding capacity, which can be advantageous for interface engineering (e.g., coatings or pads) and as a robust carrier scaffold for active agents. Nevertheless, BC and CNF systems face practical challenges related to cost and scalability, and, similar to CNC-based films, they typically require pairing with antimicrobial phases to achieve direct microbial inhibition [71,72,73].

#### 4.2.2. Chitosan

Elaborate reviews on cationic biopolymers elucidate the fundamental mechanism wherein the positively charged amino groups of chitosan engage in electrostatic interactions with the negatively charged bacterial membranes, leading to cellular destabilisation [10]. Reinforcement with organoclays or AgNPs improves the gas barrier and mechanical strength of chitosan films. Furthermore, empirical studies focused on bionanocomposite synergy demonstrate that ZnO-reinforced chitosan films provide a substantial synergistic effect, extending the commercial shelf life of poultry meat [16,61]. In parallel, investigations into complex ternary systems (PVA/Starch/Chitosan) have explored the integration of NiO-CuO nanoparticles, confirming their capacity to impart superior mechanical properties to the composite film [74]. Nonetheless, the major drawback for high aw meat systems is that biopolymer films can suffer from moisture-driven property loss and require compatibilisation, crosslinking, or multilayer designs to achieve industrially stable barrier performance—steps that may increase processing complexity and cost.

#### 4.2.3. Pectin and Pullulan

Innovative research regarding sustainable barrier films reports the development of pectin matrices incorporating green synthesised ZnO NPs (derived from tomato and passion fruit extracts), which resulted in improved UV barrier performance [75]. These films significantly reduced *Enterobacteriaceae* growth on refrigerated chicken meat. Similarly, the fabrication of pullulan/chitosan composite films reinforced with ZnO NPs synthesised via Enoki mushroom extract and propolis has been documented, demonstrating remarkable antioxidant and antibacterial activity against pork meat [76].

#### 4.2.4. Thermoplastic Starch (TPS)

Investigations into biodegradable thermoplastic matrices explored starch-based films infused with iron oxide (FeO) and ZnO nanoparticles, revealing that the amorphous nature of the starch matrix effectively masks the crystalline character of the nanoparticles, thereby influencing their diffusion kinetics [77].

#### 4.2.5. Emerging Systems

Carbon nanotubes (CNTs) and mesoporous silicon nanoparticles are being investigated as reservoirs for the slow release of essential oils [78,79].

#### 4.2.6. Green Synthesis and Microfabrication

Conventional physical and chemical methods for obtaining nanoparticles often involve toxic solvents and high energy consumption. In contrast, researchers in nanobiotechnology [5,9] propose using biological systems—especially fungi—as “bionanofactories”. Mycofabrication offers distinct advantages: fungi secrete large amounts of extracellular enzymes and proteins that act simultaneously as reducing agents (converting metal ions into nanoparticles) and as stabilising agents (‘capping agents’), preventing agglomeration.

##### Fungus-Mediated Synthesis

The synthesis of silver nanoparticles (AgNPs) using filtrates from *Aspergillus oryzae* and *Penicillium* spp. has been described [5]. These particles, ranging in size from 8 to 17 nm, have demonstrated exceptional stability and potent antimicrobial activity. The fungal proteins coating the nanoparticles improve their biocompatibility and can enhance their antibacterial activity.

##### Bacteria-Mediated Synthesis

In the realm of probiotic-mediated nanotechnology, studies utilising the strain Lactobacillus plantarum TA4 for the biosynthesis of zinc oxide nanoparticles (ZnO NPs) have yielded particles with a spherical/oval morphology and an average size of 29.7 nm [13]. This research demonstrated that biosynthesised ZnO NPs release Zn^2+^ ions at a significantly higher rate than bulk zinc oxide, a physicochemical attribute that explains their superior antimicrobial efficacy.

##### Plant Extracts

Comprehensive reviews on green chemistry methodologies advocate for the utilisation of extracts derived from fruit peels, tea leaves, or medicinal plants to reduce metal ions. This approach is not only eco-friendly but also facilitates the valorisation of agricultural by-products, aligning with the principles of the circular economy [8].

### 4.3. Beyond Migration: Cost, Environmental Footprint, and Consumer Acceptance (Critical Implementation Constraints)

While migration remains a central regulatory criterion, the feasibility of nano-enabled antimicrobial packaging is jointly constrained by technoeconomic scalability, life-cycle and end-of-life impacts, and consumer trust. These dimensions are frequently underreported in efficacy-focused studies, yet they determine whether a nanomaterial moves from laboratory validation to manufacturable products [67].

#### 4.3.1. Technoeconomic Viability and Scale-Up Burden

Commercial adoption in meat supply chains is strongly shaped by ‘shelf-life benefit per unit cost’. Beyond the price of the nanomaterial itself, additional costs arise from dispersion control (to avoid aggregation and performance variability), tighter process monitoring, and expanded compliance testing and documentation for food contact authorisation. Commercial analyses of controlled-release active packaging emphasise that these manufacturing and validation overheads can outweigh technical gains unless the target product category has a sufficient value margin or the design delivers clearly measurable risk reduction [51,80].

#### 4.3.2. Environmental Trade-Offs and End-of-Life Complexity

Nanotechnology is often framed as a sustainability lever via food waste reduction, yet environmental performance must be defended through life-cycle reasoning rather than assumed. Prospective LCAs indicate that nanoparticle synthesis routes and upstream energy/reagent inputs can dominate cradle-to-gate impacts for some systems (e.g., AgNPs), making ‘green’ synthesis beneficial only when it materially reduces these burdens at relevant scales [17]. Moreover, embedding persistent inorganic phases into polymer matrices can complicate recycling and, for biodegradable matrices, may influence biological end-of-life pathways; therefore, designing for circularity (mono-material structures where possible, minimised additive complexity, and clear end-of-life scenarios) should be treated as a first-order constraint [81,82].

#### 4.3.3. Consumer Acceptance, Perceived Exposure, and Communication

Empirical evidence indicates that public acceptance of nanotechnology is highly sensitive to affect and trust, and nano-enabled packaging is generally perceived as more acceptable than nano-enabled foods; nevertheless, willingness to purchase depends on whether the consumer perceives a concrete benefit that outweighs perceived health risks [54]. Systematic syntheses further show that acceptance is higher for ‘informational’ smart packaging functions (freshness indicators) than for systems perceived as releasing substances into food, underscoring the strategic value of low-migration or contact-active designs accompanied by transparent communication and governance [83].

### 4.4. Mechanisms of Antimicrobial Action

In food, nanomaterials are most often used in active packaging films/coatings, edible coatings, or (less commonly) as direct additives to inhibit spoilage and pathogens.

The main mechanisms by which nanomaterials exert their antimicrobial properties against food pathogens and microorganisms in general are summarised in Table 5.

An important aspect that prevents the development of bacterial resistance to nanoparticles is the fact that multiple simultaneous antimicrobial mechanisms are used by each type of nanomaterial, a behaviour that could explain their efficiency even in the case of multidrug-resistant species. Although their precise mechanisms are not fully understood, some main antibacterial pathways have been recognised.

A detailed analysis of the literature reveals multiple and often synergistic mechanisms by which nanoparticles inhibit microorganisms:

#### 4.4.1. Cell Envelope Binding and Membrane Disruption (Contact-Killing)

Many bacterial cell surfaces are net negatively charged, so nanoparticles (especially those with cationic surfaces) can electrostatically bind to the cell wall/membrane.

This binding can destabilise membrane structure, increase permeability, and cause leakage of intracellular contents, ultimately leading to loss of viability. Chitosan-based systems are a classic example where electrostatic interactions contribute strongly to membrane destabilisation (including interactions with LPS in Gram-negative bacteria). In packaging, this is often designed as a surface-mediated effect (“contact active”) to aim to kill the food packaging interface while minimising nanoparticle migration into the food [20,84].

In practical meat-packaging conditions, the efficacy of purely surface-mediated “contact-killing” mechanisms may be significantly constrained by the physicochemical nature of the meat surface itself. Fresh meat surfaces are intrinsically heterogeneous and microscopically rough, being rapidly covered by an exudate layer composed of water, proteins, lipids, and soluble metabolites released post-mortem. This biological conditioning film can act as a diffusion barrier that partially screens direct interactions between immobilised nanoparticles in the packaging matrix and bacterial cells, thereby reducing the probability of sustained nanoparticle–membrane contact [85]. Recent studies on antimicrobial active packaging emphasise that protein-rich exudates and surface moisture may adsorb onto functionalised polymer films, forming an interfacial layer that attenuates electrostatic interactions and limits the accessibility of contact-active antimicrobial sites [85,86].

Consequently, under real food contact conditions, antimicrobial performance is often governed by a combined mechanism in which limited contact-mediated disruption is complemented by the controlled release of metal ions or reactive species from the packaging surface. Contemporary research highlights that ion-mediated activity (e.g., Ag^+^ or Zn^2+^ release) can dominate over strict contact-killing when a conditioning organic layer develops at the food packaging interface, enabling antimicrobial action even without direct nanoparticle–bacteria collision [87]. This dual-mechanism paradigm—surface contact coupled with low-level ion migration is increasingly recognised as a critical design consideration for nano-enabled antimicrobial packaging intended for high-moisture, protein-rich foods such as fresh meat. Therefore, the real-world effectiveness of contact-active films should be interpreted not as a purely diffusion-free surface phenomenon but as a dynamic interplay between interfacial fouling, nanoparticle immobilisation, and controlled ion release under refrigerated storage conditions [85,86,87].

#### 4.4.2. Physical/Mechanical Damage to the Cell Membrane

Certain carbon-based nanomaterials (notably graphene oxide/graphene-based sheets) and nanofibers or needle-like morphologies (material-dependent) can exert membrane stress via sharp edges, direct contact damage, or “wrapping” effects that isolate the cell from its environment, promoting leakage and loss of integrity. These effects are most relevant when materials are immobilised in surfaces/films, where contact with cells is likely. This is typically a contact surface mechanism [20,88,89].

Advanced imaging analyses utilising scanning electron microscopy (SEM) presented in recent experimental studies [13] and described [48] in systematic reviews show severe distortions of the bacterial cell membrane. This leads to increased permeability and leakage of intracellular contents (proteins and reducing sugars), a phenomenon confirmed by biochemical tests.

#### 4.4.3. Reactive Oxygen Species (ROS) Generation

The antimicrobial effect of nanomaterials can occur from generating reactive oxygen species (ROS) such as superoxide (O_2_•−), hydroxyl radicals (•OH), and hydrogen peroxide (H_2_O_2_). Although microorganisms have antioxidant defences, ROS can exceed their capacity and can induce lipid peroxidation, loss of proteins/enzyme function, damage to nucleic acids and cell death. This mechanism is commonly encountered in metal-based nanopaticles such as TiO_2_ nanoparticles, ZnO nanoparticles, CuO, Fe-based materials, and Ag nanoparticles [12,90,91,92]. Quantitative assays performed in vitro have quantified reactive oxygen species (ROS) production in bacterial cells treated with ZnO NPs, demonstrating a direct correlation between ROS levels and cell death [13].

ROS generation is influenced in general by size, morphology and formulation of nanopaticles; in food systems, mechanisms depend also heavily on light exposure, oxygen availability, humidity, and whether the nanoparticle surface is blocked by food components [93,94].

#### 4.4.4. Release of Antimicrobial Metal Ions (“Ion Toxicity”)

Also, materials that contain metal-based nanoparticles could partially dissolve and release ions, e.g., Ag^+^, Zn^2+^, and Cu^2+^ that have toxic effects on microorganisms. They can bind to thiol (-SH) groups in proteins and lead to enzyme inactivation; can disrupt electron transport and conduct ATP depletion; could interfere with membrane proteins and transporters; trigger oxidative stress indirectly; and inhibit DNA replication/repair.

Elaborate mechanistic discussions explain that the released Ag^+^ or Zn^2+^ ions interact with the thiol (-SH) groups of respiratory enzymes and nucleic acids, blocking replication and energy metabolism [56].

In food, ion release is affected by pH, salt/ionic strength, organic acids, and proteins. Many foods can reduce effective ion activity by binding ions (complexation) [95].

#### 4.4.5. Intracellular Penetration and Interference with DNA/Protein Synthesis

After membrane perturbation, nanomaterials or released ions can trigger DNA damage, disrupt replication/repair, and inactivate proteins—often as part of a broader oxidative and membrane-disruption cascade. Intracellular mechanisms often become dominant when there is a sufficient exposure dose at the microbial surface and/or significant ion release in the local microenvironment (e.g., at the packaging interface). In general, many nanomaterials have a multi-target mode of action. In the case of silver nanoparticles, for example, literature commonly describes action mechanisms such as oxidative stress, protein dysfunction, membrane disruption, and DNA damage (Figure 2) [90,92,95].

#### 4.4.6. Local Microenvironment Changes (pH, Redox, Dehydration)

Certain oxides can shift the local environment. For example, MgO can create a more alkaline microenvironment near the surface (a thin surface water layer with higher pH), which can destabilise membranes and contribute to cell death [96].

Nanocomposites can also reduce surface water activity or alter local redox conditions, indirectly stressing microorganisms. These effects tend to be surface-local and depend strongly on moisture.

#### 4.4.7. Biofilm Inhibition and Anti-Adhesion Effects

**Figure 3:**
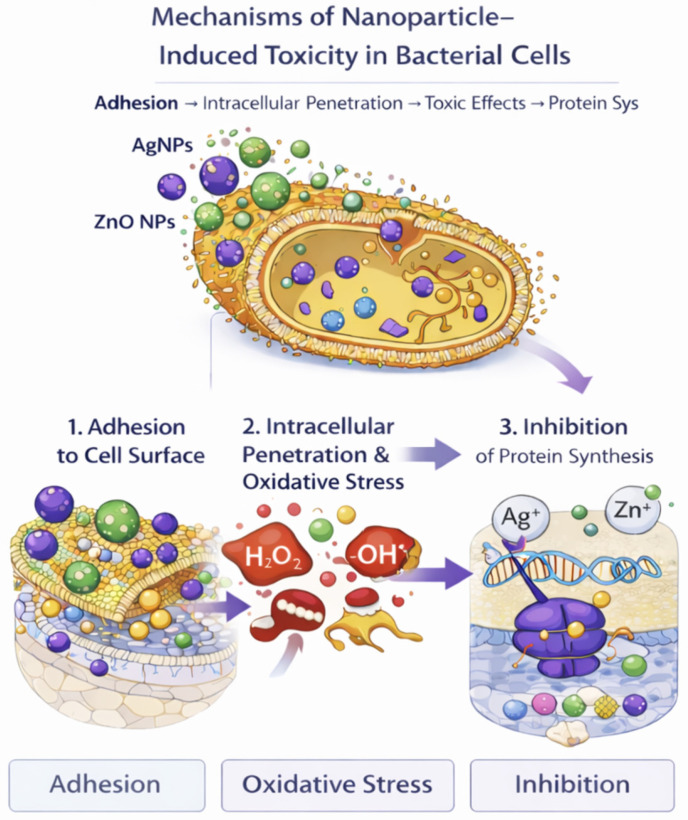
Molecular mechanisms of nanoparticle toxicity in bacterial cells. The figure schematically illustrates the sequence of interaction stages considered in the methodological assessment of metal-based nanoparticle effects on bacterial cells. These stages include: (1) adhesion to the bacterial cell surface, mediated by physicochemical interactions between nanoparticles (e.g., AgNPs and ZnO NPs) and components of the bacterial cell wall and membrane; (2) intracellular penetration and induction of oxidative stress, associated with the generation of reactive oxygen species (ROS), such as hydrogen peroxide (H_2_O_2_) and hydroxyl radicals (•OH), and disruption of cellular redox homeostasis; and (3) inhibition of protein synthesis, attributed to interactions of internalised nanoparticles or released metal ions (Ag^+^ and Zn^2+^) with intracellular targets, including ribosomal structures and nucleic acids. This schematic representation was employed as a methodological reference for the organisation and evaluation of experimental assays related to membrane integrity, oxidative stress, and protein biosynthesis (created in BioRender.com, accessed on 5 February 2026).

Biofilms can form on many surfaces found in the food industry, including stainless steel surfaces. Their presence in food systems represents a huge problem for food safety and quality, as they can be a source of pathogenic and spoilage microorganisms [97]. Nanomaterials can prevent microorganisms from establishing biofilms by reducing initial adhesion (surface energy/roughness effects), disrupting extracellular polymeric substances (EPS), interfering with quorum sensing (signalling), and killing early colonisers at the surface (Figure 3).

According to recent advances in nanocomposite applications, graphene oxide nanosheets decorated with silver nanoparticles presented antibiofilm capacity against *P. aeruginosa* [20]. Also, blended PLGA/chitosan electrospun nanofibers functionalised with hybrid graphene/silver nanostructures were able to inactivate *E. coli*, *P. aeruginosa* and *S. aureus* upon direct contact with bacterial cells, proving the capacity to inhibit microbial growth on solid surfaces and biofilm development [20].

## 5. Recent Nano-Applications in Meat

While the primary focus of this section is on widely consumed meat categories with major industrial relevance (poultry, beef, and pork), selected niche products such as rabbit meat and fish are also briefly discussed. These matrices represent underexplored but technologically relevant systems that may exhibit distinct physicochemical properties, spoilage pathways, and packaging requirements compared to conventional meat products. Their inclusion therefore aims to provide a more comprehensive perspective on nano-enabled antimicrobial packaging, particularly for emerging or less-investigated applications that could require tailored preservation strategies. In this context, fish products are considered within the broader framework of animal-derived muscle foods commonly addressed in antimicrobial packaging research, rather than being a primary focus of the review. The validation of technologies requires demonstrating their effectiveness in real food matrices. Recent studies (2014–2025) demonstrate their potential to extend shelf life and ensure meat safety, providing convincing evidence of nanocomposites’ performance.

### 5.1. Applications in Poultry Meat (Chicken and Turkey)

Poultry meat is highly perishable and susceptible to the presence of critical pathogens.

#### 5.1.1. *Campylobacter* Control

Experimental data [13] showed that biosynthesised ZnO NPs are highly effective against *Salmonella*, *E. coli*, and *S. aureus* isolated from chicken, destroying bacterial biofilms and inducing cell death through oxidative stress. Other researchers [24] developed absorbent pads and 3D tubes with immobilised ZnO nanoparticles. Tests on raw chicken meat showed a significant reduction in the *C. jejuni* population via ROS generation and the degradation of bacterial macromolecules, without affecting sensory quality.

#### 5.1.2. Polymer-Metal Synergy

Studies on eco-friendly bionanocomposites [16] used chitosan films with ZnO to control spoilage microbiota in fresh poultry meat, observing enzyme inhibition and extended shelf life. Another study used a PVA-CNCs-AgNPs composite on chicken breast, demonstrating potent inhibition against *S. aureus* and *E. coli*, with silver migration below EFSA limits [7]. Investigations into intelligent packaging demonstrated a Gellan gum and AgNPs-based H_2_S sensor that changes colour in correlation with the spoilage of chicken and fish, providing a non-invasive method of verification [34].

#### 5.1.3. Edible Coatings

Investigations into protein-based matrices [98] applied an edible coating based on *Vicia villosa* protein isolate with ZnO to refrigerated chicken breast, successfully maintaining texture and extending freshness. In a significant study focusing on sustainable bionanocomposites, pectin films with ZnO NPs (obtained from passion fruit extract) were applied to refrigerated chicken fillets [75]. The results showed a significant reduction in the Enterobacteriaceae population and the TVB-N (total volatile nitrogen) index, a marker of protein spoilage. However, the authors noted an increase in lipid oxidation (TBARS) in some cases, suggesting a possible pro-oxidant effect of certain plant extracts that requires optimisation.

Taken together, these studies indicate that nano-enabled systems applied to poultry meat consistently combine multiple antimicrobial mechanisms, including ROS generation, membrane disruption, and, in some cases, controlled ion release, leading to significant reductions in key pathogens such as *Campylobacter*, *Salmonella*, and *E. Coli* [7,24]. The convergence of results across films, pads, and edible coatings suggests that polymer–metal nanocomposites are particularly effective in high-moisture matrices such as poultry, where rapid microbial growth is the primary spoilage driver [16,98]. However, some formulations also showed secondary effects, such as increased lipid oxidation or matrix-dependent variability, highlighting the need for careful optimisation of nanoparticle loading, dispersion, and interaction with protein-rich exudates [98]. Overall, poultry applications demonstrate that nano-enabled packaging can simultaneously enhance microbial safety and shelf-life stability, provided that oxidative side effects and migration aspects are adequately controlled [7,16,24].

### 5.2. Applications on Red Meat (Beef, Rabbit, Lamb)

In red meat, preventing lipid and myoglobin oxidation is crucial.

#### 5.2.1. Rabbit Meat

Recent developments in active biogenic composites report the creation of a biodegradable film derived from berry wax and AgNPs [64]. The film reduced lipid oxidation indices (TBARS) and volatile nitrogen (TVB-N) while simultaneously inhibiting *E. coli and S. aureus.*

#### 5.2.2. Lamb Meat

Studies utilising “one-pot” fabrication techniques created a CS/PVA/AgNP film through green synthesis [99]. Applied to lamb meat, it kept the microbial load below the spoilage limit for 30 days, compared to only 6 days for the control, demonstrating exceptional preservation capacity.

#### 5.2.3. Beef

Reviews on active and intelligent packaging describe the use of smart indicators that change colour in the presence of spoilage metabolites, providing consumers with a visual guarantee of beef safety [4]. The use of natural extracts (e.g., rosemary and oregano) in combination with nanocarriers has demonstrated the ability to extend the shelf life of minced meat and sausages, reducing the need for synthetic nitrites, a significant health concern.

Collectively, applications on red meat reveal a slightly different functional emphasis compared to poultry systems, with a stronger focus on controlling lipid oxidation and preserving colour stability alongside microbial inhibition [64,99]. The reviewed studies consistently show that AgNP- and ZnO-based bionanocomposites can reduce TBARS and TVB-N indices while maintaining microbial loads below spoilage thresholds, thereby addressing both oxidative and microbiological deterioration pathways typical of beef, lamb, and rabbit meat [64,99]. These findings highlight the multifunctional role of nano-enabled films, which act not only as antimicrobial barriers but also as modulators of oxidative reactions affecting myoglobin and lipid fractions [4]. Nevertheless, the variability between animal species and fat content underscores that the effectiveness of nanocomposites is highly matrix-dependent, requiring tailored design strategies for different red meat systems [4,64].

### 5.3. Applications on Fish (Cod)

Fish products present unique challenges related to their delicate texture and rapid enzymatic degradation. Comparative study of ZnO vs. polylysine: A comparison of the effects of packaging containing nano-ZnO or polylysine on the microbial purity and texture of cod fillets yielded remarkable results [27]. The results were remarkable: nano-ZnO packaging best maintained the fish’s texture (reducing gumminess and water loss) and was more effective at inhibiting psychrotrophic and mesophilic bacteria than polylysine. This demonstrates the superiority of inorganic nanomaterials in maintaining the structural integrity of fish muscle tissue.

Overall, studies on fish packaging confirm the high sensitivity of aquatic muscle tissue to enzymatic degradation, moisture loss, and psychrotrophic bacterial growth, making it a stringent test matrix for nano-enabled packaging [100,101]. The comparative evidence indicates that inorganic nanomaterials, particularly ZnO-based systems, are capable of simultaneously maintaining microbial purity and preserving textural integrity, outperforming some conventional antimicrobial agents such as polylysine [101,102]. This suggests that, beyond antimicrobial action, nanocomposite packaging can influence water retention and structural stability of fish muscle, which are critical quality parameters for seafood products [100,102].

## 6. Limitation and Challenges

Despite the remarkable progress in nanostructured packaging, persistent challenges should be considered when using nanoparticles for the food sector. On the one hand, the effectiveness of metal nanoparticles is superior to that of classical additives. Their mechanisms of action (oxidative stress, membrane disruption) make it difficult for bacteria to develop resistance. The multifunctionality of materials (e.g., ZnO providing simultaneous antimicrobial and UV barrier protection) and AgNPs’ ability to inhibit biofilms [7], as highlighted in comprehensive reviews of silver applications, are significant advantages that can reduce global food waste.

The green synthesis (microfabrication and use of plant extracts) proposed by pioneers in nanobiotechnology [5,9] addresses the residual toxicity of chemical solvents. However, the variability in the composition of natural extracts can lead to nanoparticles with non-uniform sizes and shapes, affecting the reproducibility of antimicrobial activity on an industrial scale. Standardisation of biosynthesis processes is a critical step.

On the other hand, analytical studies [6] highlight the risks associated with increased reactivity. The “large surface area” paradox means that particles lethal to bacteria can also be toxic to human cells. Most studies are laboratory-scale, lacking data on the behaviour of nanomaterials under real logistical conditions (thermal fluctuations and mechanical shocks) that could accelerate migration. Furthermore, interaction with food additives in processed products has been poorly investigated. A promising direction is the development of “responsive” packaging [49] which releases active agents only when signs of spoilage (changes in pH or gases) are detected, minimising consumer exposure. Although many studies show that migration is often below legal limits, the variability of results requires standardisation of testing protocols. Furthermore, discrepancies between FDA and EFSA regulations create trade barriers.

In terms of effectiveness, recent experimental evidence [77], ref [1] confirms that active packaging can significantly extend the shelf life of meat products. However, comprehensive assessments of food safety management systems [23] warn that even a high-performance system supported by advanced technology may be insufficient if the raw material has a very high initial pathogen load (e.g., *Campylobacter*), emphasising the need for an integrated ‘farm-to-fork’ approach. Another important aspect refers to migration of the package materials into the food and exposure of the population to different quantities of nanomaterials that could accumulate in time. For this reason, it is very important to know very well the properties of the nanomaterials, their biocompatibility and biodegradability. For packaging systems that rely on migrating antimicrobial compounds (including nanomaterials or nano formulations), the primary challenge is demonstrating controlled and safe migration into food (or proving non-migration if that is the design intent). EFSA’s nano risk-assessment framework requires particle characterisation and exposure assessment for food-chain uses (including food contact materials) [103].

The correct evaluation of the efficiency of the packaging should also be rigorously considered. Sometimes efficacy drops in real meat matrices. Antimicrobial compounds that perform well in lab media may underperform on meat because of fat/protein binding, high ionic strength, and surface topography [104,105]. On the other hand, nano- or additive-loaded films can suffer from dispersion/aggregation, variable performance, and manufacturability issues at industrial line speeds. This aspect is more important considering that many active systems add cost and complexity to products, and, in some cases, deliver only marginal benefit if the product already respects strong direction (good hygiene + tight cold chain + optimised modified atmosphere packaging). Considering the increase in the value of the finished product, the benefit obtained from using a certain type of packaging must be carefully evaluated [106,107].

Meat packaging often needs high-barrier multilayers; these are hard to recycle, and adding active components can further complicate recyclability and food contact compliance. Recent reviews discuss sustainability pressure alongside performance requirements as a defining commercialisation challenge [108,109].

Also, consumers want “fresh-looking” meat, long shelf life, and fewer additives—but may be sceptical of “nano” or “chemical” packaging. Communicating benefits without triggering perceived risk is a real commercialisation hurdle [106].

## 7. Conclusions

Antimicrobial nanomaterials are redefining food safety paradigms in the meat industry, providing powerful tools to combat pathogens and reduce food waste. This review paper highlights four main conclusions to help guide further research in the field of nanostructured materials in the meat industry:

1. Efficacy: AgNP- and ZnO-based nanocomposites, integrated into biopolymer matrices (chitosan, pectin, and pullulan), demonstrate superior antimicrobial activity against *Salmonella*, *Campylobacter*, *E. coli* and *S. aureus*, extending the shelf life of refrigerated meat.
2. Sustainability: biological synthesis of nanoparticles (using fungi, bacteria or plants) represents the sustainable direction for the future, eliminating toxic reagents and providing materials with increased biocompatibility.
3. Integrated Intelligence: packaging is no longer just a passive barrier; the integration of nanosensors allows for active monitoring of freshness, increasing consumer confidence.
4. The Imperative of Regulation and Research: large-scale commercial success depends on harmonising migration testing standards and conducting long-term toxicological studies. The industry must adopt a precautionary approach, validating the safety of each new nanomaterial under real-world conditions of use to protect public health and ensure consumer acceptance. The future belongs to intelligent systems and responsive materials that optimise the risk-benefit balance.

## Figures and Tables

**Table 1:** Comparative analysis of methods for detecting microbial contamination in meat products.

Type of Method	Principle of Operation	Target Indicators/Mechanism	Advantages/Limitations in the Context of Meat	Reference
**Classic Microbiological Methods**	Cultivation on selective media and colony counting (CFU).	Specific pathogens (*Salmonella*, *Campylobacter*) and TVC (Total Viable Count).	**Advantages:** Gold standard for accuracy and legislative validation. **Limitations:** Long response time (24–72 h); destructive; requires a laboratory.	**[33]**
**Nanostructured Colorimetric Sensors**	Colour change of nanoparticles (e.g., AgNPs) through chemical reaction with spoilage gases.	Hydrogen sulphide (H_2_S), volatile amines (TVB-N). E.g.: AgNPs (yellow) → Ag_2_S (colourless/brown).	**Advantages:** Real-time monitoring; non-destructive; visible to the consumer. **Limitations:** Sensitivity dependent on gas concentration in the packaging.	**[34]**
**Smart pH indicators**	Colour variation of natural (anthocyanins) or synthetic dyes depends on the pH of the environment.	Increase in pH caused by the accumulation of ammonia and biogenic amines in meat.	**Advantages:** Biocompatible (if natural); low cost; integration into film. **Limitations:** May be influenced by moisture in fresh meat packaging.	**[35]**
**Time-Temperature (TTI) Sensors**	Enzymatic reactions, polymerisation or diffusion influenced by thermal history.	Monitoring of cold chain interruption (critical factor for meat).	**Advantages:** Correlates thermal abuse with estimated bacterial growth; easy to read. **Limitations:** Does not detect direct contamination but rather favourable conditions; risk of false-negative results.	**[6]**

**Table 2:** Comparison of EU and US regulatory approaches regarding migration limits and authorisation frameworks for silver (Ag) and zinc oxide (ZnO) in food contact materials.

Substance	EU (EFSA/EU Plastics Regulation)	USA (FDA Framework)	Reference
Silver (Ag)	SML: 0.05 mg Ag/kg food (substance-specific authorisations; ion migration limits in defined applications)	No harmonised SML; evaluated case-by-case via Food Contact Notification (FCN) and exposure assessment	EU: [42]; US: [57].
Zinc oxide (ZnO, expressed as Zn)	SML: 25 mg Zn/kg food (migration expressed as total zinc)	No harmonised SML; authorisation based on intended use, exposure estimation, and toxicological data	EU: [58]; US: [57,59]

Abbreviations: SML—Specific Migration Limit; EFSA—European Food Safety Authority; FDA—U.S. Food and Drug Administration; FCN—Food Contact Notification. Note: EU limits refer to migration expressed as elemental silver or zinc where applicable. The US framework relies on substance-specific authorisations and exposure-based safety evaluation rather than universally defined SML values.

**Table 3:** Summary of the effectiveness of nanomaterials organised by material type (material first).

Nanomaterial	Key Properties	Specific Applications in Meat	Predominant Mechanism	References
**AgNPs (Silver)**	Thermal stability, broad spectrum, and high surface area/volume ratio.	Films for chicken and rabbit meat; H_2_S sensors for fish.	Oxidative stress (ROS), binding to thiol (-SH) groups, and DNA disruption.	[7]
**ZnO (Zinc Oxide)**	UV blocking, biocompatibility (GRAS), and low cost.	Absorbent pads for chicken; chitosan-ZnO composite films.	Photocatalysis (H_2_O_2_ generation) and cell wall destruction.	[12,24]
**Chitosan**	Biodegradable, cationic, and bioadhesive.	Matrix for Ag/ZnO in poultry meat packaging.	Electrostatic interaction with bacterial membrane and barrier effect.	[16,61]
**Nanocellulose**	Mechanical reinforcement and carrier for controlled release.	Biodegradable films for processed meat.	Improved oxygen and water barrier and sustained ion release.	[62,63]

**Table 4:** Summary of nanomaterial efficacy organised by target microorganism (microorganism first).

Target Microorganism	Relevant Meat Type	Effective Nanomaterial	Result Observed in Studies	References
***Campylobacter jejuni***	Raw chicken meat	**ZnO NPs (immobilised)**	Significant reduction in population in exudate; maintenance of sensory quality.	[24]
***Salmonella* spp.**	Poultry meat, processed	**AgNPs**, **ZnO**	Inhibition of biofilm formation; penetration of the exopolysaccharide matrix.	[25,26]
***E. coli/S. aureus***	Beef, pork, and chicken	**AgNPs** (Green Synthesis), **PVA Composites**	Extensive inhibition zones; reduction in total microbial load (TVC).	[7,64]
**Alteration microbiota** (Psychrotrophs)	Fish and chilled meat	**Chitosan + ZnO/Ag**	Extension *of shelf life*; reduction in lipid oxidation rate (TBARS).	[16,28]

**Table 5:** Dominant antimicrobial drivers in nanomaterials have recently been investigated for the food industry.

Nanomaterial (Food Context)	Dominant Antimicrobial Drivers
Metal-based nanoparticles (AgNPs, ZnO, TiO_2,_ and CuO)	Ion release and oxidative stress
Chitosan nanoparticles	Electrostatic binding to cell wall, membrane permeabilisation, and chelation
Nanoemulsions/encapsulated essential oils	Improved dispersion and sustained release; primarily membrane disruption of bacteria/fungi
Nanocomposite films (e.g., polymer + nanoparticles)	Controlled release and/or contact-killing at the packaging surface; often biofilm inhibition

## Data Availability

No new data were created or analyzed in this study.
